# Effects of Wine Components in Inflammatory Bowel Diseases

**DOI:** 10.3390/molecules26195891

**Published:** 2021-09-28

**Authors:** Josip Vrdoljak, Marko Kumric, Tina Ticinovic Kurir, Ivan Males, Dinko Martinovic, Marino Vilovic, Josko Bozic

**Affiliations:** 1 Department of Pathophysiology, University of Split School of Medicine, 21000 Split, Croatia; josip.vrdoljak@mefst.hr (J.V.); marko.kumric@mefst.hr (M.K.); tticinov@mefst.hr (T.T.K.); dinko.martinovic@mefst.hr (D.M.); marino.vilovic@mefst.hr (M.V.); 2 Department of Endocrinology, Diabetes and Metabolic Diseases, University Hospital of Split, 21000 Split, Croatia; 3 Department of Surgery, University Hospital of Split, 21000 Split, Croatia; ivmales@kbsplit.hr

**Keywords:** wine, inflammatory bowel disease, resveratrol, polyphenols, Crohn’s disease, ulcerative colitis, diet, inflammation

## Abstract

With the rising prevalence of Inflammatory bowel disease (IBD) worldwide, and the rising cost of treatment with novel biological drugs, there is an increasing interest in various diets and natural foods as a potential way to control/modulate IBD. As recent data indicates that diet can modify the metabolic responses essential for the resolution of inflammation, and as wine compounds have been shown to provide substantial anti-inflammatory effect, in this review we aimed to discuss the current evidence concerning the impact of biological compounds present in wine on IBD. A number of preclinical studies brought forth strong evidence on the mechanisms by which molecules in wine, such as resveratrol or piceatannol, provide their anti-inflammatory, anti-oxidative, anti-tumor, and microbiota-modulation effects. However, concerning the effects of alcohol, it is still unclear how the amount of ethanol ingested within the framework of moderate wine consumption (1–2 glasses a day) affects patients with IBD, as human studies regarding the effects of wine on patients with IBD are scarce. Nevertheless, available evidence justifies the conductance of large-scale RCT trials on human subjects that will finally elucidate whether wine can offer real benefits to the IBD population.

## 1. Introduction

Inflammatory bowel disease (IBD) consists of a spectrum of chronic, non-communicable, multifactorial diseases of the gastrointestinal tract. Two main types of IBD are Crohn´s disease (CD) and ulcerative colitis (CD) [1]. The global prevalence of IBD is rising, increasing the load on the working population and the healthcare system [2,3,4]. There are a lot of unknowns in the etiology and pathophysiology of IBD. It is considered that IBD occurs in a complex interplay of susceptible genes, an ill-fitted diet, changed intestinal microbiota, and a pathologic immune response to dietary elements and intestinal microbes [5,6,7]. The current treatment modalities for IBD consist of anti-inflammatory drugs (salicylates etc.), immunosuppressants (azathioprine, corticosteroids, etc.), biological medications (anti-TNF-α, anti-integrin, cytokine-targeted therapy), and surgical treatment [8,9,10]. Besides these therapeutic modalities, there are also dietary therapies such as Exclusive Enteral Nutrition [11,12]. In addition, there is an increasing interest in various diets and natural foods as a potential way to control/modulate IBD [13]. The usual suspects being the Paleolithic diet, low-FODMAP diet, gluten-free diet, specific carbohydrate-based diet, and the Mediterranean diet (MedDiet) [12,13,14]. Interestingly, recent data indicates that diet can modify the metabolic responses essential for the optimal healing of injury-induced inflammation, as nutrients can act as signaling agents [14]. Lately, the MedDiet, primarily through the usage of red wine and olive oil, is coming to the forefront of prevention and management of chronic diseases, including cardiovascular disease (CVD), diabetes mellitus type 2, cancer, and IBD [15,16,17].

The Mediterranean diet is a dietary pattern commonly found in the olive oil tree-growing parts of the Mediterranean basin [15,18]. Red wine and olive oil have been a central part of the Mediterranean diet since the days of the ancient Greeks and Romans. Even since those days, people have talked about the health benefits of olive oil and wine, but only now, in modern times, can we dissect these foods and look at the molecules that give them the desired effects on health.

This review will discuss the current evidence concerning the impact of biological compounds found in wine on IBD. We will describe the composition of wine and the impacts of wine as a whole or its isolated compounds on IBD pathophysiology by reviewing in vitro, animal, and human studies, whilst respecting that a limited amount of these compounds are found in wine.

### 1.1. Wine Composition

Wine, one of the oldest alcoholic beverages, is created during a process of grape must fermentation. The main constituents of wine are water, ethanol (usually between 9–15%), carbohydrates, organic acids (malic acid, citric acid, tartaric acid, etc.), as well as polyphenols, and volatile compounds [19,20]. The technological process of winemaking and the different types of grapes used offer a plethora of different wines with varying levels of alcohol and polyphenols [19,21]. Specifically, the concentration of phenolic compounds within grapes is dependent on grape variety, growing season, soil type, maturity of wine, as well as environmental and climatic conditions [22]. The phenolic composition (both concentration and composition) changes mainly in the first steps of vinification and continues during storage. In line with this, it is important to address that phenolic composition of the final wine differs from the composition of the corresponding grapes as a consequence of production of new derivatives, such as tyrosol, flavenes, and free phenolic acids [23]. The total content of phenolic compounds in grapes is affected by several factors: cultivar, the geographic origin, year of production, soil chemistry, degree of maturation, as well as solar radiation and temperature [23]. For all these reasons, estimation of polyphenol content in wine is rather challenging, and consequently, it represents a major obstacle in creation of “standardized moderate consumption” of wine.

**Figure 1:**
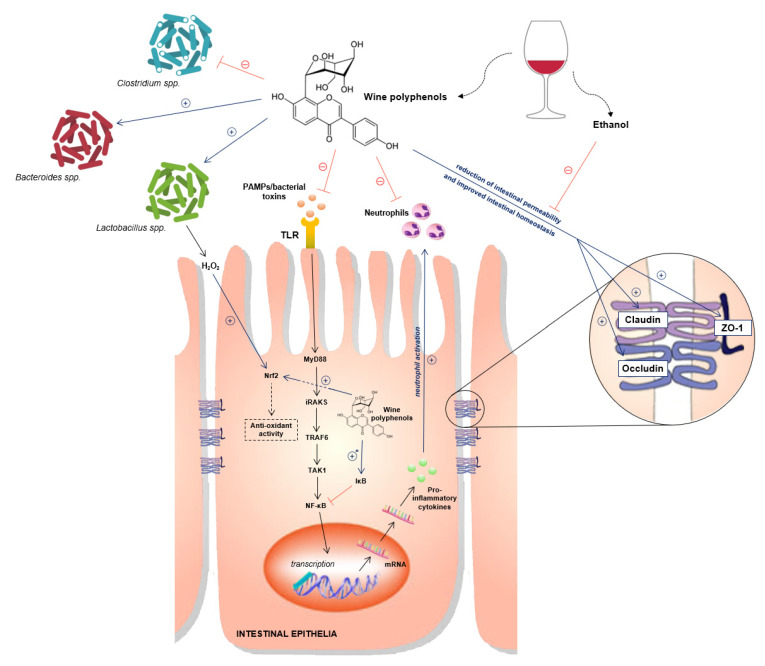
Multiple molecular targets of wine polyphenols contributing to its anti-inflammatory and anti-oxidant effects, changes in intestinal permeability and gut microbiota. Abbreviations: PAMPs: Pathogen-associated molecular patterns; ZO-1: Zonula occludens-1; TLR: Toll-like receptor; Nrf2: nuclear factor erythroid-derived 2; MyD88: Myeloid differentiation primary response 88; iRAKS: Interleukin-1 receptor associated kinase; TRAF6: Tumor necrosis factor receptor (TNFR)-associated factor 6; TAK1: transforming growth factor-β-activated kinase 1; NF-κB: nuclear factor kappa-light-chain-enhancer of activated B cells; IκB: inhibitor of nuclear factor kappa B.

Polyphenols are considered the main bioactive components in wine that positively affect health (prevention and management of non-communicable diseases) [19,24]. Because all grape parts are used during the winemaking process of red wine, the polyphenolic content is much higher in red wine when compared to white wine (1–5 g/L vs. 0.2–0.5 g/L) [18]. Polyphenols consist of a wide variety of chemical compounds that are generally classified into two main branches: flavonoids, and non-flavonoids [25]. Flavonoids are represented by flavonols (quercetin and myricetin), chalcones, flavononols, flavanols (catechin and epicatechin), flavones, anthocyanidins and isoflavonoids. Non-flavonoids are represented by phenolic acids, stilbenes (resveratrol), coumarins, lignans, and tannins [25,26]. Most of these compounds have demonstrated some or all of these desired effects: antioxidant, anti-inflammatory, anti-cancer, and anti-microbial (Figure 1) [27,28].

### 1.2. Wine Composition

Since IBD is a chronic, non-communicable, inflammatory disease, a rising number of research papers are trying to ascertain the potential positive effects of wine or its components on IBD. Many of the phenolic compounds in wine have low bioavailability, and hence, reach low concentrations in the bloodstream, while their high content present in the gut can produce a more significant effect on enterocytes and the bacterial flora [29,30].

#### 1.2.1. The Evidence In Vitro

The pathophysiology of IBD consists of an aberrant immune response of the gut, with an increased expression of pro-inflammatory cytokines and an increased creation of reactive oxygen species (ROS). This cascade’s main factors are COX-2, iNOS, IL-8, TNF-α, and NF-κB. Furthermore, a critical mechanistic determinant of IBD is a dysfunctional intestinal barrier, as seen through altered expression and subcellular distribution of tight junction (TJ) proteins [31]. Nunes et al. have demonstrated how a polyphenolic extract from Portuguese red wine decreased the paracellular permeability in cytokine-stimulated HT-29 colon epithelial cells. The red wine extract induced a significant increase in the mRNA of the barrier-forming TJ proteins occludin, claudin-5, and *zonula occludens* (ZO)-1 compared to control cells. It also led to less formation of claudin-2 mRNA, which is a channel-forming protein usually induced by pro-inflammatory conditions [31]. One other paper that focused on the impact of wine-digested fluids on gut microbiota has shown an increase in *Akkermansia*, *Selenomonadaceae*, and *Megasphaera* genus levels, as well as a positive change in short-chain fatty acid (SCFA) levels which could lead to decreased paracellular permeability [32].

On the other hand, a study by Asai et al. exhibited how a low and acute dose of ethanol leads to apoptotic cell death in confluent Caco-2 cells and, therefore, impairs intestinal barrier function [33]. Interestingly, these positive impacts of wine polyphenols on intestinal permeability in vitro are in contrast to in vivo findings by Swanson et al., where moderate wine consumption led to increased intestinal permeability [34].

Considering intestinal permeability, in vitro evidence on the impact of red wine extract and wine digested fluids suggests a protective effect on the cellular barrier [31,32]. This is in stark contrast to the in vitro evidence using an acute dose of ethanol and the in vivo evidence provided by Swanson et al. [33,34]. Hence, it is probable that the polyphenolic content per se has a positive effect on intestinal permeability, while the alcoholic content potentially negates that effect. 

Moreover, another study with Portuguese red-wine extract enriched in anthocyanins exhibited an anti-inflammatory effect in HT-29 colon epithelial cells stimulated with pro-inflammatory factors (TNF-α, IFN-γ, and IL-1). It was shown how the wine extract decreases COX-2 activity, the synthesis of iNOS and IL-8, as well as decreases the degradation of inhibitor of NF-κB [35]. Nevertheless, the polyphenol-enriched red wine extract contains a higher concentration of polyphenols than the concentration ingested in the usually recommended one glass of wine a day.

Considering the role of oxidative stress in IBD pathophysiology, many researchers have investigated the potential benefits of antioxidants. A study by Deiana et al. has shown how extracts from three different Sardinian grape varieties applied to Caco-2 cell monolayers counteracted the oxidative activity in a tert-butyl hydroperoxide (TBH)-induced oxidative damage model [36]. Furthermore, Tannin procyanidin B2 has also exhibited protective activity against oxidative stress in the human colonic Caco-2 cell model by up-regulating glutathione S-transferase P1 (GSTP1) via s ERK and p38 MAPK activation and Nrf2 translocation [37]. A study on the potential anti-oxidative effect of resveratrol on porcine intestinal-epithelial cell line (IPEC-J2) treated with deoxynivalenol (DON), has shown a reduction in ROS levels via Nrf2 signaling pathway activation [38]. Moreover, numerous studies have exhibited the anti-inflammatory effects of resveratrol in intestinal cells. In one study on Caco-2 cells exposed to bacterial lipopolysaccharide, resveratrol reduced the rate of degradation of an endogenous NF-κB inhibitor (IκB), and therefore led to a reduction of NF-κB activity with a decrease in COX-2 expression [39]. Another beneficial effect of resveratrol is on alleviating mitochondrial dysfunction [40]. When resveratrol was applied in extremely high concentrations it prevented indomethacin-induced mitochondrial dysfunction in Caco-2 cells [40,41]. In another study on Caco-2 cells stimulated with LPS, where researchers studied the effects of polyphenols from red wine, cocoa and green tea they found how a dietary dose moderately modulates intestinal inflammation, but does not increase HDL production [42].

A number of in vitro experiments using batch culture fermentation have shown beneficial effects of wine components on fecal microbiota. The common impact seen is a growth enhancement of *Bifidobacterium* spp. and *Lactobacillus* spp., with growth inhibition of the *Clostridium* group [43,44,45,46,47]. These experiments show how wine and its components have a prebiotic effect on the “good” gut bacteria while also showing anti-microbial results on those bacteria that could lead to intestinal pathology.

Overall, the above-mentioned in vitro experiments provide evidence that wine and wine polyphenols have the following benefits: (i) they decrease the activity of NF-κB and therefore decrease the production of pro-inflammatory cytokines, (ii) activate Nrf2 signalling pathway and therefore reduce ROS levels, (iii) polyphenols (quercetin) bind to the ubiquinone site of complex I protecting it from inhibitors like indomethacin and decreasing mitochondrial dysfunction, (iv) wine polyphenols support the growth of healthy microbiota and inhibit the growth of pathologic microbiota (Table 1).

#### 1.2.2. The Evidence on Animal Models

As reviewed by Nunes et al., the benefits of resveratrol, a key active molecule present in red wine, have also been confirmed in animal models of IBD and intestinal cancer [38]. For example, Martin et al. have shown that, in an early colonic inflammation model caused by trinitrobenzenesulphonic acid (TNBS) instillation in rats, resveratrol (5–10 mg/kg/day) administration has significantly decreased the index of neutrophil infiltration and levels of proinflammatory cytokine IL-1β, whilst reducing the degree of colonic injury [48]. Furthermore, the same researches later showed how resveratrol extended its benefits to a rat model of chronic gut inflammation caused by TNBS. Resveratrol treatment led to decreased neutrophil infiltration, reduced TNF-α levels, reduced COX-2 and the NF-κB p65 protein expression, and also led to a significant increase of TNBS-induced apoptosis in colonic cells [49]. Moreover, another study on the DSS-induced colitis mouse model, but with a diet enriched with resveratrol, showed attenuation of colitis signs and symptoms. The mice that ate a resveratrol enriched diet (at 20 mg/kg of diet) maintained their body weight and had less diarrhoea and rectal bleeding. All the mice in the treatment group survived compared to the 40% mortality rate in the non-resveratrol group. The same study also showed a significant reduction in proinflammatory cytokines, TNF-alpha and IL-1beta, and an increase of the anti-inflammatory cytokine IL-10 [50].

Moreover, Larrosa and associates demonstrated on rats with DSS-induced colitis how low doses of resveratrol, similar to the dosage contained in a hypothetical daily diet of a person weighing 70 kg, lead to a reduction in mucosal levels of inflammatory markers [51]. Similar to the previously mentioned studies, prostaglandin E (PGE)-2, COX-2, and PGE synthase-1 were affected. In addition, this study showed an increase in *Bifidobacterium* and *Lactobacillus* spp. with a reduction in *E. coli* growth [51]. This is in line with other studies that have shown that resveratrol enhances the growth of *Lactococcus lactis*, whilst inhibiting the growth of *Enterococcus faecalis* [52]. Importantly, the relation between resveratrol and gut microbiota is a two-way street [53]. On one hand, resveratrol modulates gut microbiota, yet on the other, resveratrol can be transformed by gut microbiota into various bioactive metabolites. 

In a DSS-induced colitis mouse model, Li et al. have exhibited that mice fed with muscadine grape phytochemicals (MGP) or muscadine wine phytochemicals (MWP) for 14 days had decreased levels of proinflammatory cytokines (IL-6, TNF-α), reduced myeloperoxidase activity, while also preventing weight loss and preserving colonic length [54]. A study researching the effects of grape seed extract (GSE) in a rat model of DSS-induced ulcerative colitis yielded promising results. Male Sprague-Dawley rats were fed daily (days 0–10) with GSE (400 mg/kg), and compared with no-GSE controls, GSE-fed rats had significantly decreased ileal villus height (14%; *p* < 0.01) and mucosal thickness (13%; *p* < 0.01), approaching the values of healthy controls. GSE also reduced the qualitative histological severity score (*p* < 0.05) in the proximal colon, but there was no significant effect in the distal colon [55]. In a study investigating the effects of proanthocyanidins from grape seed (GSPE) in a TNBS-induced recurrent ulcerative colitis rat model, Wang et al. demonstrated how GSPE treatment led to a recovery of pathologic changes in the colon, reduced the colonic weight/length ratio, and improved the macroscopic and microscopic damage scores. Furthermore, iNOS and myeloperoxidase activities were significantly reduced in the GSPE group, while the superoxide dismutase and gluthatione peroxidase activities were significantly increased [56]. 

These data indicate that proanthocyandins and wine/grape phytochemicals have similar and probably synergistic effects on the colonic mucosa. The main characteristics exhibited were modulation of the inflammatory response, inhibition of inflammatory cell infiltration, a reduction in ROS-damage, and a promotion of colonic tissue repair and regeneration [53,54,55,56].

Correspondingly, and as reviewed previously, a number of animal studies have exhibited the benefits of resveratrol in decreasing the risk of colon cancer in animal models with chronic intestinal inflammation [40]. In one study on a DSS colitis mouse model, they found how resveratrol inhibited the formation of polyps, and also reduced cell damage and subsequent proliferation of epithelial cells in the intestinal mucosa [57]. By these effects, resveratrol inhibited the tumour initiation process, and we can argue that by these mechanisms it can potentially lead to a decrease in colon cancer incidence in chronic intestinal inflammation, such as IBD-. Moreover, in a study by Cui et al., the authors demonstrated that resveratrol reduces tumour incidence and tumour multiplicity. In mice treated with azoxymethane (AOM) + DSS, tumour incidence was 80%, while the mice treated with AOM + DSS + resveratrol (300 ppm) had a 20% tumour incidence. Moreover, AOM + DSS-treated mice had 2.4 +/−0.7 tumours per animal, while the resveratrol treated group had 0.2 +/− 0.13 tumours per animal [58]. In another study, rats were fed with processed meat, and red wine and pomegranate extracts were added to their diet. The rats that were fed polyphenols had significantly less precancerous lesions, with a full suppression of faecal excretion of nitrosyl iron, therefore suggesting that nitrozation could be a promoter of carcinogenesis [59].

Additionally, Dolara et al. have showed how red wine polyphenols can influence carcinogenesis, intestinal microflora, oxidative damage and gene expression profiles in rats [60]. The rats were treated with Azoxymethane (AOM) and 1,2-dimethylhydrazine dihydrochloride (DMH) for colon cancer/adenoma induction, and statistically significant reduction in adenoma number was seen in DMH group, while a significant reduction in total tumor number (cancer + adenoma) was seen in AOM group. What’s more, the main bacterial strains in the polyphenol treated group were *Bacteroides*, *Lactobacillus* and *Bifidobacterium* spp., whereas in the control group the predominant strains were *Bacteroides*, *Clostridium* and *Propionibacterium* spp. [60]. The authors used wine polyphenols which contained 4.4% anthocyanins, 0.8% flavonols, 2.0% phenolic acids, 1.4% catechin, 1.0% epicatechin and 28.0% proanthocyanidin units, consisting of 18.0% epigallocatechin, 13.2% catechin, 65.0% epicatechin and 3.8% epicatechin gallate [59]. In addition, in a study by Femia et al., the authors researched the effects of red wine polyphenols on AOM-induced colon carcinogenesis in rats [61]. The results showed that rats treated with total polyphenolic extracts from red wine (WE) had significantly less colorectal adenomas, while there was no noticeable difference in rats treated with high molecular weight polyphenols (HMWP) or low molecular weight polyphenols (LMWP), respectively, suggesting that a synergistic effect of polyphenols is needed to exert a beneficial outcome [61].

Interestingly, a recent study investigated piceatannol, an analogue of resveratrol found in grapes and wine as well, different just by an additional hydroxyl group located at the 3′-carbon and metabolically more stable than resveratrol [61,62,63]. They showed how piceatannol significantly inhibited VEGF-mediated signalling and cell proliferation in VEGF-treated colon cancer cells (HT-29), as well as suppressed VEGF-mediated angiogenesis in zebrafish embryos [64]. Piceatannol is abundant in wine, with an average concentration of 13.1 ng/mL in French red wine (3× that of resveratrol) [65]. Furthermore, as it was shown that piceatannol has a higher oral bioavailability than resveratrol, added that it is also more metabolically stable than resveratrol, whilst having similar anti-inflammatory, anti-cancer, and cardioprotective properties, piceatannol positioned itself as a molecule on which additional future research should be focused [61,64,65].

Overall, the presented studies on animal models confirm the findings from in vitro studies (Table 2). These studies show how bioactive compounds found in wine (resveratrol, piceatannol, proanthocyanidins, total phenolic extracts) exert an anti-inflammatory, anti-oxidative, and anti-tumor effect, as well as positive effect on intestinal flora [39,50,51,58,59,61].

#### 1.2.3. The Evidence on Humans

While the evidence of the beneficial effects of wine and/or wine polyphenols on intestinal inflammation are plentiful in in vitro and animal models, the human in vivo studies are still lacking large randomized control trials (RCTs) and meta-analyses. Nevertheless, the current evidence is also promising and mandates further research on the matter.

An RCT study investigating the effects of resveratrol on patients with ulcerative colitis, in which the patients were given 500 mg resveratrol or placebo capsule for 6 weeks, showed that resveratrol supplementation led to a significant decrease in plasma levels of TNF-a, hs-CRP, as well as decrease in activity of NF-kB in PBMCs [66]. Moreover, the score of inflammatory bowel disease questionnaire-9 (IBDQ-9) increased, while the clinical colitis activity index score has significantly decreased in the treatment group [66]. In a small sample study by Sabzevary-Ghahfarokhi et al., it was demonstrated on patients with UC that resveratrol can reverse the inflammatory effects of TNF-α by reducing IL-1β and increasing IL-11 production, thereby providing protective effects on UC patients [67]. Additionally, a study by Gonzalez et al. analyzed intestinal immune markers in healthy volunteers before and after red wine consumption. They demonstrated that in a subgroup of participants with a high basal cytokine level, red wine ingestion led to a significant reduction in pro-inflammatory markers (TNF-α, IL-6, and IFN-γ) that usually promote initial inflammation [68]. However, poor water solubility and low bioavailability of resveratrol limit its clinical applications [69]. Hence, Intagliata et al. recently reported multiple modalities that could reverse these issues using different delivery systems such as liposomes, polymeric and lipid nanoparticles, but also by chemical modifications thus improving its physicochemical properties [70].

Furthermore, the aforementioned study by Swanson et al. investigated the effects of moderate red wine consumption on intestinal permeability and stool calprotectin, which are associated with recurrent IBD disease activity [34]. Interestingly, the study had mixed results, as 1–3 glasses of daily red wine consumption led to decreased stool calprotectin levels in inactive IBD patients, while it also led to increased intestinal permeability measured by urinary lactulose/mannitol excretion (small bowl permeability) and urinary sucralose secretion (large bowl permeability). Nevertheless, the study had several notable limitations: small sample size (21 subjects), short follow-up duration (1 week), and a lack of assessment of mucosal activity. We can argue that these effects result from harmful effects of alcohol on those areas of the gut that are sensitive because of previous inflammatory damage and match the location of the disease. Given all the evidence from in vitro and animal studies regarding the anti-inflammatory effects from compounds found in wine, we hypothesize how these biologically active compounds are causing the decrease in stool calprotectin levels. In a previous study, the same author found how patients with inactive IBD drink alcohol in quantities similar to the general population, and how 75% of IBD patients reported a worsening of GI symptoms after drinking alcohol [71]. Another prospective cohort study also indicated how alcohol consumption increases the risk of exacerbation in patients with UC [72].

Moreover, in a crossover study, Hey et al. investigated the effects of five different alcoholic drinks on patients with CD [73]. Twenty patients with CD in remission and twelve healthy controls were randomly given red wine, white wine, Smirnoff Ice, Elephant Beer and pure ethanol. No differences in alcohol absorption were found between the groups, but CD patients reported a higher abdominal pain symptom score after ingesting Smirnoff Ice and Elephant beer. Authors argue how high sugar content present in these drinks leads to more intestinal fermentation that could present itself with symptoms of abdominal pain and bloating [73].

In our previous observational study investigating MedDiet adherence in patients with IBD, only 4.5% of patients in the UC group and 8% of patients in the CD group reported daily red wine intake in the MedDiet framework. Notably, when the participants were asked about the suspected foods that aggravate IBD-related symptoms, 50% in the UC group and 36% in the CD group reported alcohol as a suspect [74]. Interestingly, in a Swedish prospective cohort study that showed how the MedDiet lowers the risk of late-onset Crohn’s disease, moderate alcohol intake increased with the MedDiet adherence score. In the highest MedDiet score bracket [6,7,8], 61% of participants moderately consumed alcohol, while the number of moderate alcohol consumers in the lowest bracket [0–2] was only 14% [75]. While some of the IBD patients associate wine intake with symptom aggravation, it seems that moderate wine consumption in the framework of the MedDiet could lead to IBD prevention [74,75]. 

## 2. Precautions and Future Directions

In general, alcohol was shown to increase gut permeability by causing transepithelial and paracellular permeability [76]. Chronic alcohol ingestion also leads to gut dysbiosis (less *Lactobacillus* and *Bifidobacterium* spp.), bacterial overgrowth, and a disruption of intestinal immune response [76,77,78]. The research shows that chronic and uncontrolled ethanol ingestion disrupts intestinal homeostasis and increases intestinal and, later on, systemic inflammation [76,79]. The other well-established deleterious effects of wine on liver function, metabolism, brain, and alcohol addiction, must not be neglected as well [80]. On the other hand, as we have discussed in this review, other studies show how biologically active compounds found in alcoholic beverages such as wine (polyphenols, tannins, organic acids) have a completely different effect on intestinal homeostasis, and exert anti-inflammatory, anti-oxidative, and positive microbiota effects, making wine capable of assisting in disease control and affecting disease monitoring [29,40]. Nevertheless, most of the alcohol-mediated effects seem to aggravate intestinal inflammation and consequently impact disease onset, recurrence, and control of symptoms. Furthermore, British Society of Gastroenterology consensus guidelines address the importance of alcohol reduction because alcohol further reduces bone mineral density, which is already substantially struck by corticosteroids [81]. Finally, alcohol use interferes with the metabolism of most IBD medications, leading to either increase in side effect occurrence rate or loss of drug’s effect [82]. Specifically, mesalamine, azathioprine, methotrexate, and biologic medications can all be affected by concomitant alcohol intake via a variety of mechanisms. Nevertheless, a large number of authors advocates moderate wine consumption based on inferences drawn from large-scale populational studies. Although questioned by certain authors, the J-shaped curve explaining the relationship between alcohol use and total mortality has been well established [83,84]. Namely, the lowest mortality risk was observed at 6 g/day of alcohol (half of a drink/day), but with lower mortality with up to 4 drinks/day in men and 2 drinks/day in women when compared with no alcohol consumption, even after adjustment for a myriad of confounding variables. Furthermore, as presented by Xi et al., light alcohol consumption appears to be protective against cancer mortality, unlike heavy alcohol use which is associated with increased cancer risk [85,86,87]. Unlike the cardiovascular effects of wine, which have been extensively studied, the role of wine in IBD, or any other gastrointestinal pathology for that matter, has been poorly elucidated. Although certain inferences from studies exploiting the effects of wine on vascular function can be drawn to IBD because of the overlapping mechanisms, such as anti-inflammatory properties and protection from oxidative stress, the evidence on the effect of wine and alcohol on IBD course is still inconclusive. 

Hence, as detrimental effects seem to prevail, at least for now, future research should focus on finding the optimal dose of red wine for these patients. We are casting about for dosage (if it exists) in which the beneficial effects of polyphenols, tannins, and organic acids will outweigh the detrimental effects of ethanol. It should also be noted that even if we found optimal dosage, adherence to the exact dosage of wine will be very challenging, markedly owing to the addictive nature of alcohol consumption. In summary, before we have firm evidence from more extensive prospective studies, caution should be advised in recommending red wine consumption to patients with IBD.

## 3. Conclusions

This review summarized the current evidence concerning the effects of wine compounds on IBD. A number of in vitro and animal model studies provide strong evidence on the mechanisms by which molecules found in wine, such as resveratrol or piceatannol provide their anti-inflammatory, anti-oxidative, anti-tumor, and microbiota-modulation effects. However, concerning the effects of alcohol, it is still unclear how the amount of ethanol ingested within the framework of moderate wine consumption (1–2 glasses a day) affects patients with IBD, as human studies on the effects of wine and its molecules on IBD/intestinal inflammation are scarce. In addition, it is doubtful whether the above-noted effects can be obtained by drinking wine exclusively, as this beverage contains only scarce amount of these compounds. Since more and more patients are turning to dietary options, such as the Mediterranean diet, as a means to control their diseases, there is an increasing need for high-quality, evidence-based information. Therefore, we have a strong foundation for translation into clinical studies and human research. With the rising prevalence of IBD worldwide and the rising cost of treatment with novel biological drugs, wine polyphenols could serve as a cheaper therapeutic modality accessible to more patients. We conclude that the evidence provided can serve as a basis for large-scale RCT trials on human subjects that will finally elucidate whether wine can offer real benefits to the IBD population.

## Figures and Tables

**Table 1:** In vitro studies on wine polyphenols and intestinal inflammation.

Study	Cell Type	Intervention	Results
Nunes et al. [31]	Cytokine-stimulated HT-29 colon epithelial cells	Polyphenolic extract from Portuguese red wine	↑ mRNA of TJ-proteins ↓ mRNA of channel forming proteins
Zorraquín-Peña et al. [32]	Caco-2 cell monolayers grown in Transwell^®^ inserts	Intestinal-digested wine (IDW) and colonic-digested wine (CDW)	Reduction in *Bacteroides* and an increase in *Veillonella, Escherichia/Shigella* and *Akkermansia.* ↑ SCFA levels
Nunes et al. [35]	HT-29 colon epithelial cells stimulated with pro-inflammatory factors	Portuguese red-wine extract enriched in anthocyanins	↓ degradation of IκB ↓ COX2 ↓ iNOS ↓ Interleukin 8
Deiana et al. [36]	Caco-2 cell monolayers stimulated with tert-butyl hydroperoxide	Wine extracts from three different Sardinian grape varieties	Scavenging of reactive oxygen species and/or prevention of their formation
Rodriguez-Ramiro et al. [37]	Human colonic Caco-2 cell model	Tannin procyanidin B2	Up-regulation of glutathione S-transferase P1 (GSTP1)
Yang et al. [38]	porcine intestinal-epithelial cell line (IPEC-J2) treated with deoxynivalenol	Resveratrol	Reduction in ROS levels via Nrf2 signaling pathway activation
Cianciulli et al. [39]	Caco-2 cells exposed to bacterial lipopolysaccharide	Resveratrol	↓ degradation of IκB
Carrasco-Pozo et al. [41]	Caco-2 cells exposed to indomethacin	Quercetin, resveratrol, rutin and epigallocatechin gallate	Reduction of mitochondrial dysfunction
Nicod et al. [42]	Caco-2 cells stimulated with LPS	Polyphenols from red wine, cocoa and green tea	Moderate reduction in intestinal inflammation markers
Hidalgo et al. [43]	pH-controlled, stirred, batch-culture fermentation system	Anthocyanins and gallic acid	↑ *Bifidobacterium* spp. ↑ *Lactobacillus−Enterococcus* spp.
Cueva et al. [44]	Faecal batch-culture fermentation	Two purified fractions from grape seed extract (GSE): GSE-M (70% monomers and 28% procyanidins) and GSE-O (21% monomers and 78% procyanidins)	↑ *Lactobacillus/Enterococcus* ↓ *Clostridium histolyticum*

**Table 2:** Animal studies on wine polyphenols and IBD.

Study	Animal Model	Intervention	Results
Martin et al. [48]	TNBS instillation in rats	resveratrol (5–10 mg/kg/day)	↓ neutrophil infiltration ↓ Interleukin-1β
Martin et al. [49]	rat model of chronic gut inflammation caused by TNBS	resveratrol 10 mg/kg/day	↓ TNF-α ↓ COX 2 ↓ NF-κBp65 protein expression
Sánchez-Fidalgo et al. [50]	DSS-induced colitis mouse model	Diet enriched with resveratrol (20 mg/kg of diet)	↓ rectal bleeding ↓ diarrhoea ↓ mortality
Larrosa et al. [51]	DSS-induced colitis rat model	diet with resveratrol, similar to dosage contained in a hypothetical daily diet of a person weighing 70 kg	↓ PGE-2 ↓ COX-2 ↓ PGE synthase-1 ↑ *Bifidobacterium* and *Lactobacillus* spp. *↓* *E. coli*
Qiao et al. [52]	mice fed with high fat diet	diet with resveratrol (200 mg/kg)	↑ *Lactococcus lactis* ↓ *Enterococcus faecalis*
Li et al. [54]	DSS-induced colitis mouse model	diet with MGP or MWP	↓ Interleukin-6, TNF-α ↓ myeloperoxidase activity ↓ weight loss
Cheah et al. [55]	DSS-induced rat model of ulcerative colitis	grape seed extract (400 mg/kg) gavage	↓ ileal villus height ↓ mucosal thickness ↓ proximal colon qualitative histological severity score
Wang et al. [56]	TNBS-induced recurrent ulcerative colitis rat model	proanthocyanidins from grape seed (GSPE)	↓ colonic weight/length ratio ↓ iNOS and myeloperoxidase activity
Altamemi et al. [57]	DSS colitis mouse model	resveratrol treatment via oral gavage	↓ formation of polyps ↓ cell damage and subsequent proliferation of epithelial cells
Cui et al. [58]	mice treated with AOM + DSS,	resveratrol (300 ppm)	↓ reduces tumour incidence and tumour multiplicity
Bastide et al. [59]	rats were fed with processed meat	diet with red wine and pomegranate extracts	↓ less precancerous lesions ↓ faecal excretion of nitrosyl iron
Dolara et al. [60]	rats were treated with AOM and DMH for colon cancer/adenoma induction	diet enriched with red wine polyphenols	↓ adenoma number in DMH group ↓ total tumor number in AOM group
Femia et al. [61]	AOM-induced colon carcinogenesis in rats	rats treated with WE, HMWP or LMWP	↓ less colorectal adenomas in WE group ↔ No noticeable difference in HMWP and LMWP groups
Kwon et al. [64]	VEGF-treated colon cancer cells (HT-29), and VEGF-mediated angiogenesis in zebrafish embryos	treatment with piceatannol	↓ cell proliferation ↓ VEGF induced angiogenesis

## Data Availability

Not applicable.

## Abbreviations

TJ-protein	Tight Junction protein
SCFA	Short-chain fatty acid
COX2	Cyclooxygenase-2
I-κB	inhibitor of nuclear factor kappa B
iNOS	Inducible nitric oxide synthase
LPS	Lipopolysaccharides
TNBS	trinitrobenzenesulphonic acid
WE	total polyphenolic extracts from red wine
HMWP	high molecular weight polyphenols
LMWP	low molecular weight polyphenols
MGP	muscadine grape phytochemicals
MWP	muscadine wine phytochemicals
VEGF	Vascular endothelial growth factor
DSS	dextran sulfate sodium
AOM	azoxymethane
DMH	1,2-dimethylhydrazine dihydrochloride
PGE2	Prostaglandin E_2_
TNF-α	tumor necrosis factor alpha
